# Comparative Transcriptome Analysis Reveals the Seawater Adaptation Mechanism in *Pseudaspius hakonensis*

**DOI:** 10.3390/genes17010076

**Published:** 2026-01-09

**Authors:** Ziyue Xu, Wen Zheng, Wenjun Chen, Min Zhou, Dongdong Zhai, Ming Xia, Hongyan Liu, Fei Xiong, Ying Wang

**Affiliations:** 1 Hubei Engineering Research Center for Protection and Utilization of Special Biological Resources in the Hanjiang River Basin, School of Life Sciences, Jianghan University, Wuhan 430056, China; beckyxuziyue20@163.com (Z.X.); 15902793284@163.com (W.Z.); zhoumin340@163.com (M.Z.); zhaidongdong@jhun.edu.cn (D.Z.); xm1990schaoren@163.com (M.X.); lhy9603@126.com (H.L.); xf9603@163.com (F.X.); 2 State Key Laboratory of Breeding Biotechnology and Sustainable Aquaculture, Institute of Hydrobiology, Chinese Academy of Sciences, Wuhan 430072, China; wenjunchen0423@gmail.com

**Keywords:** *P. hakonensis*, comparative transcriptome, seawater adaptation, osmoregulation, MAPK signaling

## Abstract

Background: The family Cyprinidae is predominantly restricted to freshwater habitats, making the evolution of diadromy and seawater adaptation exceptionally rare within this group. *Pseudaspius hakonensis*, a rare anadromous cyprinid, and its strictly freshwater congener *P. leptocephalus*, provide an ideal comparative model to investigate the molecular mechanisms underlying salinity adaptation. This study aimed to elucidate the tissue-specific transcriptional reprogramming, identify candidate genes and key pathways, and explore their association with seawater acclimation in *P. hakonensis*. Methods: We performed comparative transcriptomic analyses of gill, liver, and kidney tissues from both species using RNA-Seq. Sequencing reads were aligned to a high-quality reference genome of *P. hakonensis*. Differential expression analysis was conducted using DESeq2, followed by functional enrichment analyses (GO and KEGG) to identify significant biological processes and pathways. Results: A total of 8784, 5965, and 5719 differentially expressed genes (DEGs) were identified in gill, kidney, and liver tissues, respectively, with the gill showing the highest differences. Functional enrichment revealed tissue-specific roles: gill DEGs were associated with protein synthesis and energy metabolism; kidney DEGs with transport and detoxification; and liver DEGs with metabolic regulation and stress signaling. Cross-tissue analysis highlighted three core pathways consistently enriched: MAPK signaling, ABC transporters, and glutathione metabolism. Key candidate genes, including *DUSP10*, *SLC38A2*, *ATP8B1*, *GSTA4*, and *MGST1*, were significantly upregulated in *P. hakonensis*. Conclusions: This first multi-tissue transcriptomic comparison of an anadromous and a freshwater cyprinid reveals pervasive, tissue-specific molecular reprogramming underlying seawater adaptation in *P. hakonensis*. The coordinated activation of MAPK signaling, glutathione metabolism, and transporter pathways suggests an integrated regulatory network for osmoregulation and stress resistance. These findings provide novel insights into the genetic basis of salinity adaptation in cyprinids and identify candidate genes for future functional validation.

## 1. Introduction

Fish migration, a periodic and directional movement between distinct habitats [1,2], is a fundamental life-history strategy driven by requirements for reproduction, foraging, and environmental change [3,4,5]. Fish migration is broadly categorized based on lifelong habitat use into oceanodromy (entire life in marine waters) and potamodromy (entire life in freshwater) [6]. A distinct category, diadromy, involves regular migrations between freshwater and marine environments and is subdivided into anadromy (spawning in freshwater, growing in saltwater), catadromy (spawning in saltwater, growing in freshwater), and amphidromy (movements not directly tied to reproduction) [6,7,8]. As the largest freshwater fish family, most cyprinids are restricted to freshwater, and only a small number of species exhibit limited salinity tolerance, including idle (*Leuciscus idus*), roach (*Rutilus rutilus*), and bream (*Vimba vimba*) [9,10]. This imbalance raises a fundamental question: what molecular programs enable the few cyprinids that tolerate brackish environments to maintain osmotic homeostasis and complete anadromous life cycles?

*Pseudaspius hakonensis* provides a particularly informative system to address this question. The genus *Pseudaspius* is distributed around the Sea of Japan, including the Japanese Archipelago, Sakhalin Island, the southern Kuril Islands, the Russian Maritime Territory (Primorsky Region), and the Korean Peninsula. *P. hakonensis* is a nearshore anadromous cyprinid that primarily grows and matures in coastal marine and estuarine (brackish) habitats before ascending rivers to spawn [11,12]. Notably, *P. hakonensis*, previously classified within the genus *Tribolodon*, is now recognized as an endemic cyprinid species of the genus *Pseudaspius* [13]. Thus, *P. hakonensis* represents an ecologically rare instance of diadromy within Cyprinidae, making it a compelling model for studying salinity adaptation [14,15]. In stark contrast, its congeneric sister species, *P. leptocephalus*, is a strictly freshwater resident endemic to the Heilongjiang (Amur) River Basin [16]. This sister-species contrast offers a powerful phylogenetically controlled comparison: two closely related species share a recent common ancestor but occupy radically divergent osmotic niches, enabling us to reduce phylogenetic confounding when interrogating gene expression programs associated with seawater adaption. Together, this pair represents a rare natural experiment for dissecting the molecular basis of a major ecological transition within Cyprinidae.

Comparative studies of osmoregulation between marine, brackish, and freshwater fish populations have a long tradition in ichthyology, offering critical insights into physiological adaptation [17]. With the advent of high-throughput omics, high-throughput RNA sequencing (RNA-Seq) and comparative transcriptomics have become indispensable tools for elucidating the molecular basis of complex traits like osmoregulation. This approach is widely used to explore the genetic basis for salinity tolerance and osmotic regulation [18,19,20]. For example, a comparative transcriptomic approach has been used to investigate how Atlantic salmon (*Salmo salar*) adapts to varying salinity environments through the cortisol signaling pathway [21]. Similarly, comparative transcriptomic analysis of gill cells from Japanese eel (*Anguilla japonica*) under different salinity adaptations identified and localized specific immune-related genes that exhibit expression changes associated with salinity adaptation [22]. Furthermore, multi-tissue transcriptomic analyses coupled with qPCR validation have been employed to decipher the adaptive mechanisms of non-native minnows to river salinization [23]. Transcriptomic analyses has also been broadly applied to investigate the comprehensive physiological responses of fish gills to salinity stress. Specifically, transcriptomic sequencing was used to investigate gene expression changes in the gills of Siberian sturgeon (*Acipenser baeri*) under high salinity stress, thereby delineating the osmoregulatory mechanisms underlying their high salinity tolerance [24].

In this study, we performed comparative transcriptome analyses of gill, kidney, and liver tissues to identify DEGs, which were subsequently subjected to Gene Ontology (GO) and Kyoto Encyclopedia of Genes and Genomes (KEGG) enrichment analyses to highlight candidate genes and key pathways associated with seawater acclimation. Furthermore, we delineate expression changes in critical genes and pathways during adaptation and clarify how these molecular responses interact with high saline stress, thereby enhancing our understanding of fish osmoregulation at the molecular level and providing valuable genetic resources for research on aquatic adaptation. Through multi-tissue comparative transcriptomics, we aimed to identify core genes and pathways underpinning osmoregulatory plasticity and construct an integrative model of systemic adaptation to salinity.

## 2. Materials and Methods

### 2.1. Sample Collection, RNA Extraction and Sequencing

Adult male and healthy anadromous *P. hakonensis* was captured using gill nets from Suifen River in Dongning City, Heilongjiang Province, China. Adult male and healthy *P. leptocephalus* was obtained from the cultured stock maintained at the Heilongjiang River Fisheries Research Institute in Harbin, Heilongjiang Province, China. The *P. leptocephalus* individuals were reared in freshwater recirculating systems under controlled conditions prior to sampling. All experiments performed in this study were approved by the Institutional Animal care and Use Committee of Institute of Hydrobiology, Chinese Academy of Sciences (IHB, CAS, Protocol NO. IHB/LL/2024075). To minimize distress, all fish were euthanized with MS-222, following an approved procedure. Liver, kidney, and gill tissues were dissected immediately after euthanasia. All tissue samples for RNA extraction were flash-frozen in liquid nitrogen and stored at −80 °C until further processing. Three biological replicates (individual fish) per species per tissue were used for RNA sequencing and subsequent analysis. Total RNA was isolated using TRIzol reagent (Invitrogen, Carlsbad, CA, USA) according to the manufacturer’s protocol. RNA quality was assessed through multiple methods: integrity was examined by 1% agarose gel electrophoresis; purity and concentration were measured using a NanoDrop ND-1000 spectrophotometer (Labtech, Palaiseau, France) and a Qubit 4.0 fluorometer (Thermo Fisher Scientific, Waltham, MA, USA); and the RNA Integrity Number (RIN) was determined using an Agilent 2100 Bioanalyzer (Agilent Technologies, Waldbronn, Germany). Only RNA samples with a RIN ≥ 7.0 were used for subsequent library construction. Following quality control, mRNA was enriched, fragmented, and used to construct paired-end cDNA libraries. These libraries were sequenced on an Illumina HiSeq X Ten platform, generating 150-bp paired-end reads.

### 2.2. Sequencing Data Processing, Read Mapping and Transcriptome Assembly

Raw sequencing reads were first processed using fastp (v0.23.4) to perform quality control, including removal of adapter sequences and low-quality bases (parameters: -q 20 -u 30 —detect_adapter_for_pe) [25]. The resulting clean reads were then mapped to the high-quality, chromosome-level reference genome of *P. hakonensis* (BioProject: PRJNA980574) using the splice-aware aligner HISAT2 (v2.2.1) with parameters: —dta -p 8 —max-intronlen 5000 [26]. The *P. hakonensis* genome was chosen as the common reference for both species due to its superior assembly quality (contig N50 > 3.6 Mb) and the high degree of genomic synteny and close phylogenetic relationship with *P. leptocephalus*. This strategy ensures high mapping efficiency for both species while minimizing reference bias in the comparative transcriptomic analysis. The resulting Sequence Alignment (SAM) files were converted to sorted, indexed Binary Alignment (BAM) files using SAMtools (v1.9) [27]. Transcript assembly and quantification were performed using StringTie (v2.2.1) in two steps [28]. First, transcripts were assembled for each sample individually with guidance from the reference annotation (parameter: -G). Subsequently, to generate a unified transcriptome for consistent cross-sample expression quantification, the individual transcript files (in GTF format) were merged using StringTie’s —merge function. Finally, a gene-level read count matrix was generated from the merged transcriptome using the prepDE.py script distributed with the StringTie package. This count matrix served as the direct input for subsequent differential expression analysis. Key mapping statistics, including the total number of clean reads and the percentage of reads uniquely mapped to the reference genome for each library, are provided in Supplementary Table S1.

### 2.3. Differential Gene Expression Analysis

The analysis was performed using the DESeq2 package (v1.34.0) [29] in R. A DESeqDataSet was created from the raw read count matrix and sample metadata. The data were normalized using DESeq2’s default median-of-ratios method to account for differences in library size and RNA composition. Differential expression testing was conducted separately for each tissue (gill, liver, and kidney) to compare *P. hakonensis* and *P. leptocephalus* within the same tissue type. Genes with an adjusted P-value (false discovery rate, FDR) ≤ 0.05 and an absolute log_2_ fold change (|log_2_FC|) ≥ 1 were defined as statistically significant DEGs.

### 2.4. Functional Annotation and Enrichment Analysis

A non-redundant set of unigenes was defined by selecting the longest transcript isoform per gene locus from the merged transcriptome assembly generated by StringTie (v2.2.1). These unigenes were functionally annotated using BLAST2GO (v5.3) against several databases: Gene Ontology (GO), Kyoto Encyclopedia of Genes and Genomes (KEGG), and eggNOG (v5.0) [30,31,32]. For functional annotation in BLAST2GO, a BLASTP search (e-value ≤ 1 × 10^−5^, minimum percent identity ≥ 40%) was performed against the eggNOG (v5.0) database to assign functional annotations. Subsequently, GO functional classification was conducted for the annotated transcripts, categorizing genes by molecular function, biological process, and cellular component. To identify biological pathways significantly associated with salinity adaptation, enrichment analysis was performed on the DEGs using KOBAS software (v3.03) against the KEGG pathway database [33]. The enrichment significance was assessed using Fisher’s exact test, the resulting P-values adjusted for multiple testing via the Benjamini–Hochberg method to control the FDR. Pathways meeting a threshold of FDR-adjusted *p*-value ≤ 0.05 were considered statistically significant.

## 3. Results

### 3.1. Overview of High-Throughput Sequencing Data

**Figure 1:**
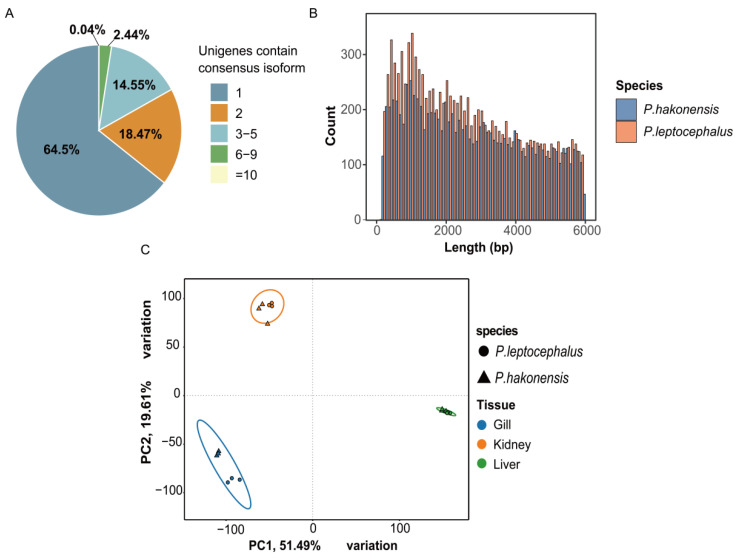
Characterization of transcriptomes and sample relationships in *P. hakonensis* and *P. leptocephalus* transcriptomes. (**A**) Number of unigenes containing consensus isoforms. (**B**) Length distribution of assembled transcripts for *P. hakonensis* and *P. leptocephalus*. (**C**) Principal components 1 and 2 of genes expressed in three tissues of gills, kidneys, and liver of *P. hakonensis* (triangles) and *P. leptocephalus* (circles).

To investigate the transcriptomic landscapes and adaptive mechanisms of *P. hakonensis* and *P. leptocephalus*, RNA sequencing was performed on gill, kidney, and liver tissues. Approximately 135 GB of high-quality clean data (71.7 GB for *P. hakonensis* and 63.3 GB for *P. leptocephalus*), with an overall average Q30 score of 93.89%, was generated. Alignment to the *P. hakonensis* reference genome (PRJNA980574) achieved an average mapping rate of 94.69% for *P. hakonensis* samples and 83.58% for *P. leptocephalus* samples, confirming the data’s high quality and suitability for subsequent differential expression analysis (Supplementary Table S1). Reference-guided transcriptome assembly identified 96,861 expressed transcripts (isoforms) across both species (Figure 1A). These isoforms originated from distinct genetic loci and exhibited a conserved length distribution, with a predominance of sequences longer than 1000 bp (Figure 1B). Comprehensive assembly metrics are summarized in Supplementary Table S2. For clarity, unigene here denotes the single, longest transcript isoform assembled for each gene locus, and DEGs were identified from the read counts summed at that unigene level.

### 3.2. Exploration of Sample Relationships

Principal Component Analysis (PCA) was performed on normalized gene expression data to elucidate sample relationships. The first two principal components (PC1 and PC2) revealed distinct clustering primarily by tissue type (gill, kidney, liver), with biological replicates from the same tissue grouping together (Figure 1C). The primary clustering of samples by tissue type (gill, kidney, liver) along PC1 and PC2, with tight grouping of biological replicates within each tissue, demonstrates that tissue-specific expression patterns constitute the major source of transcriptomic variance in this dataset [34]. Within each tissue cloud, a separation between *P. hakonensis* (triangles) and *P. leptocephalus* (circles). can be observed, with the greatest differences seen in gill samples.

### 3.3. Analysis of DEGs Among Three Tissues

**Figure 2:**
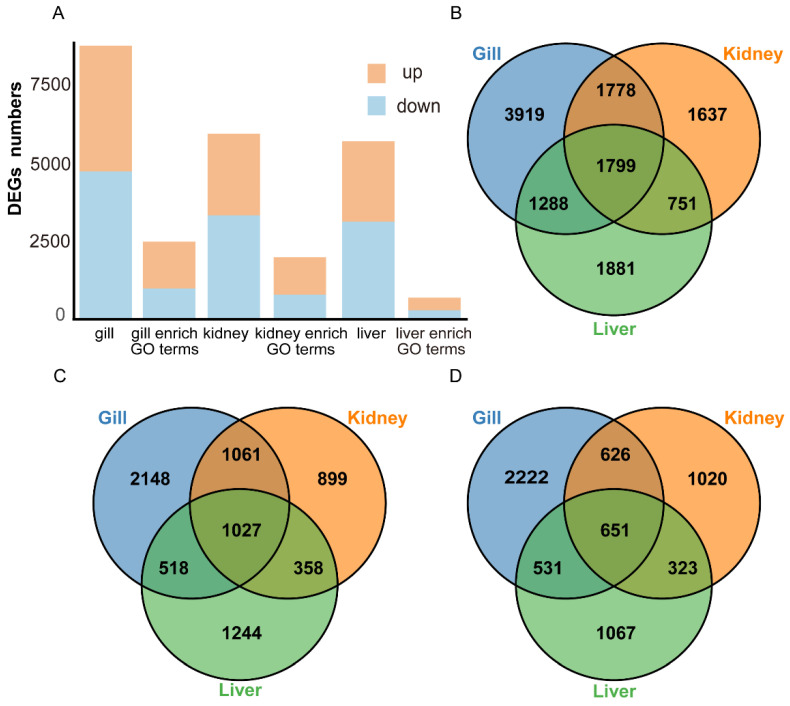
Overview of DEGs across three tissues. (**A**) Distribution of DEGs identified in the three tissues and their enrichment in specific GO terms; yellow and blue colors represent up-regulated and down-regulated DEGs, respectively. (**B**) Venn diagram illustrating the intersection of DEGs among the three tissues. (**C**) Venn diagram showing the overlap of up-regulated DEGs. (**D**) Venn diagram showing the overlap of down-regulated DEGs.

Comparative transcriptomic analysis between *P. hakonensis* and *P. leptocephalus* identified 5719, 5965, and 8784 DEGs in the liver, kidney, and gill, respectively (FDR ≤ 0.05, |log_2_FC| ≥ 1) (Figure 2A, Table 1). The gill exhibited the highest number of DEGs. In *P. hakonensis* relative to *P. leptocephalus*, 4754 (gill), 3345 (kidney), and 3147 (liver) genes were upregulated, while 4030 (gill), 2620 (kidney), and 2572 (liver) genes were downregulated (Figure 2B–D). A limited overlap of DEGs was observed across tissues (Figure 2B): 1799 DEGs were shared among all three, while the gill (3919), liver (1881), and kidney (1637) harbored distinct tissue-specific DEG subsets (Figure 2, Supplementary Table S3).

### 3.4. Functional Enrichment Analysis of DEGs

**Figure 3:**
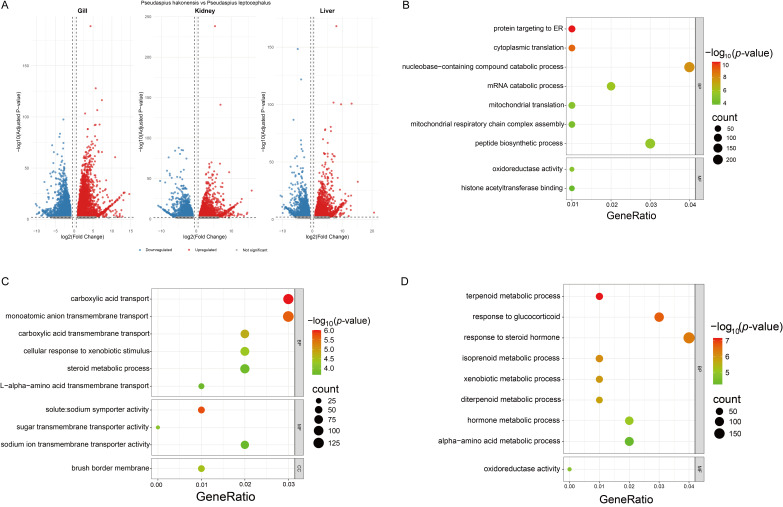
Identification of DEGs and GO functional enrichment analysis in three tissues. (**A**) Volcano plots of DEGs. Red indicates higher expression in *P. hakonensis*, blue indicates lower expression, and gray indicates no expression. (**B**) Gene Ontology (GO) enrichment analysis of DEGs in gill tissue. (**C**) GO enrichment analysis of DEGs in kidney tissue. (**D**) GO enrichment analysis of DEGs in liver tissue.

Gene Ontology (GO) and KEGG pathway enrichment analyses were performed on tissue-specific DEGs to infer their biological functions. In the gill, upregulated DEGs were significantly enriched in GO terms related to protein synthesis (e.g., “cytoplasmic translation”, “mitochondrial translation”) and KEGG pathways such as “Protein export” and “Oxidative phosphorylation”, suggesting enhanced metabolic and biosynthetic activity (Figure 3A,B). In the kidney, enriched GO terms highlighted transport and detoxification functions (e.g., “carboxylic acid transport”, “cellular response to xenobiotic stimulus”). KEGG analysis underscored roles in “Nitrogen metabolism” and “ABC transporters” (Figure 3A,C). In the Liver, Enrichment was observed in metabolic pathways including “Glycerolipid metabolism”, “Steroid biosynthesis”, and key signaling pathways like “MAPK signaling” and “Two-component system”, reflecting its central role in metabolic adjustment and stress response.

### 3.5. Integrated Pathway Analysis and Candidate Gene Expression

**Figure 4:**
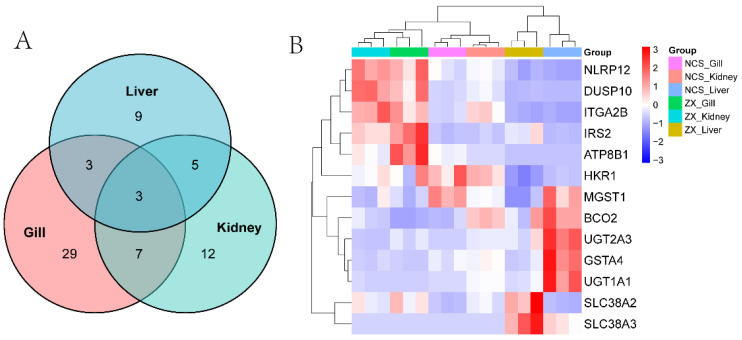
Conserved pathways and gene expression associated with seawater adaptation. (**A**) Venn diagram showing the overlap of KEGG pathways enriched in gill, kidney, and liver tissues in response to saline adaptation. (**B**) Heatmap depicting expression patterns of key genes from enriched pathways across the three tissues. Note: ZX: *P. hakonensis*; NCS: *P. leptocephalus*.

To identify systemic responses to salinity adaptation, we analyzed KEGG pathways enriched across tissues. A total of 29 pathways were commonly enriched in gill, kidney, and liver (Figure 4A). These co-enriched pathways were predominantly associated with MAPK signaling, ABC transporters, and glutathione metabolism. Heatmap visualization of gene expression within these key pathways revealed tissue-specific patterns (Figure 4B). Notable candidates included *DUSP10* (MAPK signaling negative regulator), which showed consistent upregulation across all three tissues in *P. hakonensis*. Solute carriers (e.g., *SLC38A2, SLC38A3*) and *ATP8B1* (ABC transporter) exhibited upregulated expression, particularly in gill and kidney. Glutathione metabolism genes (*GSTA4, MGST1*) were markedly elevated. NLRP12 was consistently upregulated.

### 3.6. The Schematic Diagram of Several Pathways About Seawater Adaptation

**Figure 5:**
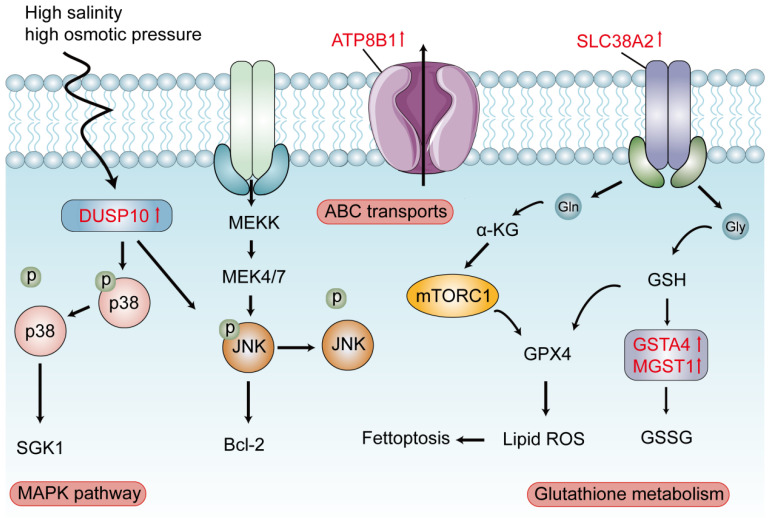
Schematic model of proposed molecular pathways involved in seawater adaptation in *P. hakonensis*. *DUSP10* (Dual Specificity Phosphatase 10), *ATP8B1* (ATPase Phospholipid Transporting 8B1), *SLC38A2* (Solute Carrier Family 38 Member 2), *GSTA4* (Glutathione S-transferase Alpha 4), *MGST1* (Microsomal Glutathione S-transferase 1). Arrows indicategene up-regulation.

We constructed a schematic diagram (Figure 5) to summarize the core molecular pathways and key genes associated with seawater adaptation. The diagram integrates three pathways consistently enriched across gill, kidney, and liver tissues: the MAPK signaling pathway, ABC transporters, and glutathione metabolism. It highlights key candidate genes significantly upregulated within these pathways. In the MAPK signaling pathway, *DUSP10* was consistently upregulated in all three tissues. Within transport-related pathways, the amino acid transporter *SLC38A2* was upregulated in the gill and kidney, and the phospholipid transporter *ATP8B1* was also upregulated. In the glutathione metabolism pathway, *GSTA4* and *MGST1* showed elevated expression. The schematic diagram presents these upregulated genes within their respective pathways and suggests potential interactions among these core pathways.

## 4. Discussion

Salinity transitions impose multifaceted challenges on fishes, requiring coordinated regulation of ion transport, epithelial barrier function, cellular stress responses, and whole-body energy allocation [35]. The sister species pair *P. hakonensis* (anadromous) and *P. leptocephalus* (freshwater resident), sharing a recent common ancestor but exhibiting divergent osmotic niches, provides a powerful natural model to disentangle the molecular mechanisms underlying seawater adaptation in Cyprinidae, a family predominantly restricted to freshwater [13]. This study presents the first multi-tissue comparative transcriptomic framework for this pair and reveals broad, tissue-dependent expression differences that are consistent with systemic remodeling in *P. hakonensis* under high-salinity ecological conditions. Our multi-tissue analysis revealed that the gill of *P. hakonensis* underwent the most extensive transcriptomic changes compared to its freshwater congener, a finding consistent with its role as the primary interface for ion and water exchange [36,37]. However, pronounced expression differences in kidney and liver indicate that salinity-associated responses are not gill-restricted but instead involve organism-wide coordination. These findings align with and extend previous transcriptomic studies on diadromous fishes. For instance, in anadromous brook charr (*Salvelinus fontinalis*), gill transcriptomes show significant remodeling related to ion transport and energy metabolism during salinity transitions [38]. Similarly, seawater-acclimated threespine stickleback (*Gasterosteus aculeatus*) exhibit upregulated gill genes related to ionocyte proliferation and ion transport, mirroring the ionoregulatory specialization observed in *P. hakonensis*. Furthermore, studies in Atlantic salmon (*Salmo salar*) have highlighted the reprogramming of gill energy metabolism during seawater adaptation [39]. Notably, we extend these consensus findings by identifying *SLC38A2* as a key upregulated solute carrier in the gill—a gene not prominently featured in the aforementioned studies. This suggests a potential cyprinid-specific integration of amino acid uptake (through *SLC38A2*) with ion gradient establishment, highlighting lineage-specific nuances in metabolic-ionic coupling [40]. Beyond the gill, the conserved enrichment of MAPK and glutathione metabolism pathways across all three tissues in our study mirrors systemic stress response networks identified in migrating salmonids [41]. This congruence across phylogenetically distant groups underscores the existence of core molecular modules for salinity adaptation, while the species-specific DEGs identified here may represent unique adaptive innovations within Cyprinidae.

Tissue-specific functional enrichment provided insights into distinct adaptive strategies. In the gill, the pronounced upregulation of genes involved in protein synthesis and ion transport (e.g., *SLC38A2*) likely supports the rapid turnover of ionocytes and the restoration of ionic balance. The kidney’s transcriptomic profile emphasized nitrogen metabolism and detoxification pathways, aligning with its critical function in ammonia excretion under high salinity. In the liver, adaptive shifts were observed in lipid metabolism and immune-related pathways (e.g., Toll-like receptor signaling), suggesting a metabolic reprioritization to meet the increased energetic demands of osmoregulation while managing systemic stress [42].

Based on the multi-tissue transcriptomic analysis, we propose an integrative model illustrating key molecular pathways implicated in the salinity adaptation of *P. hakonensis* (Figure 5). This model highlights the interplay between MAPK signaling and glutathione metabolism under osmotic stress. Key upregulated genes identified in our analysis, including the MAPK phosphatase *DUSP10*, the amino acid transporter *SLC38A2*, the phospholipid transporter *ATP8B1*, and glutathione-related enzymes *GSTA4* and *MGST1*, are positioned within their putative functional contexts [43]. The MAPK cascade, a known responder to osmotic stress [44], appears centrally regulated. The upregulation of *DUSP10* [45] suggests a potential negative feedback mechanism to fine-tune MAPK signaling and mitigate cellular stress, a response noted in other euryhaline teleosts [46,47].

Concurrently, enhanced glutathione metabolism likely plays a role in counteracting oxidative stress. Increased expression of *GSTA4* and *MGST1* could support the detoxification of lipid peroxidation products, thereby protecting against related cellular damage. The transporter *SLC38A2* [48], by facilitating glutamine uptake, may link amino acid availability to glutathione synthesis and mTORC1 signaling, integrating metabolic and stress response pathways. Furthermore, *ATP8B1* [49], an aminophospholipid transporter, might contribute to maintaining membrane asymmetry and stability under osmotic challenge [50].

A key finding is the coordinated activation of MAPK signaling, ABC transporters [51], and glutathione metabolism across all tissues, indicating a conserved regulatory network for salinity acclimation, as previously observed in other euryhaline species. Our proposed model (Figure 5) integrates MAPK signaling with antioxidant defense mechanisms [52], highlighting genes such as *DUSP10* and *GSTA4* as central players in this adaptive response. While our transcriptomic data provide correlative insights, the congruence of the enriched pathways with established osmoregulatory mechanisms strongly supports their potential functional relevance [37]. Future functional studies using gene knockdown or transgenic approaches will be essential to validate these candidate genes.

## 5. Conclusions

In conclusion, our comparative transcriptomic analysis of anadromous *P. hakonensis* and freshwater *P. leptocephalus* reveals three key findings: (1) The gill undergoes the most extensive transcriptomic changes (8784 DEGs), consistent with its role as the primary osmoregulatory organ; (2) tissue-specific adaptive strategies are evident: gill prioritizes protein synthesis and ion transport, kidney focuses on detoxification, and liver mediates metabolic adjustment; and (3) coordinated activation of MAPK signaling, ABC transporters, and glutathione metabolism forms a core regulatory network for seawater adaptation. Key candidate genes (*DUSP10*, *SLC38A2*, *ATP8B1*, *GSTA4*, *MGST1*) are identified, providing a molecular blueprint for salinity adaptation in diadromous cyprinids and targets for future functional validation.

## Figures and Tables

**Table 1:** Number of DEGs between tissues of *P. hakonensis and P. leptocephalus*.

Tissue (P. h vs. P. l)	Up-Regulated	Down-Regulated	Total DEGs
Gill	4754	4030	8784
Kidney	3345	2620	5965
Liver	3147	2572	5719
Gill vs. Kidney	1409	1410	2819
Gill vs. Liver	1607	1458	3065
Kidney vs. Liver	198	48	246

Note: P. h: *P. hakonensis*; P. l: *P. leptocephalus*.

## Data Availability

The original contributions presented in the study are included in the article. The raw sequencing data will be deposited in the NCBI Sequence Read Archive (SRA) prior to the final publication of this article. All other raw data supporting the conclusions are available from the corresponding author upon request. All other relevant data are included in the article or Supplementary Materials.

## Supplementary Materials

The following supporting information can be downloaded at: https://www.mdpi.com/article/10.3390/genes17010076/s1, Supplementary Table S1: Summary of raw sequencing metrics for *P. hakonensis* and *P. leptocephalus* samples, including read counts, base numbers, GC content, % ≥ Q30, and overall alignment rate. Supplementary Table S2. Statistics and length distribution of the de novo assembled transcriptomes for *P. hakonensis* and *P. leptocephalus*. Supplementary Table S3. The top 5 most significantly upregulated and downregulated genes in gill, kidney, and liver tissues.

## Abbreviations

The following abbreviations are used in this manuscript:

DEGs	Differentially Expressed Genes
FDR	False Discovery Rate
GO	Gene Ontology
KEGG	Kyoto Encyclopedia of Genes and Genomes
RNA-Seq	RNA Sequencing
RIN	RNA Integrity Number
SAM	Sequence Alignment/Map
BAM	Binary Alignment/Map
